# The BRG1 chromatin remodeling enzyme links cancer cell metabolism and proliferation

**DOI:** 10.18632/oncotarget.9505

**Published:** 2016-05-20

**Authors:** Qiong Wu, Pasil Madany, Jason R. Dobson, Jake M. Schnabl, Soni Sharma, Tara C. Smith, Andre J. van Wijnen, Janet L. Stein, Jane B. Lian, Gary S. Stein, Rohini Muthuswami, Anthony N. Imbalzano, Jeffrey A. Nickerson

**Affiliations:** ^1^ Department of Cell and Developmental Biology, University of Massachusetts Medical School, Worcester, MA, USA; ^2^ School of Life Sciences, Jawaharlal Nehru University, New Delhi, Delhi, India; ^3^ Department of Biochemistry and Molecular Biology, Mayo Clinic, Rochester, MN, USA; ^4^ Department of Biochemistry and Vermont Cancer Center for Basic and Translational Research, University of Vermont College of Medicine, Burlington, WA, USA

**Keywords:** breast cancer, metabolism, lipogenesis, gene regulation, BRG1

## Abstract

Cancer cells reprogram cellular metabolism to meet the demands of growth. Identification of the regulatory machinery that regulates cancer-specific metabolic changes may open new avenues for anti-cancer therapeutics. The epigenetic regulator BRG1 is a catalytic ATPase for some mammalian SWI/SNF chromatin remodeling enzymes. BRG1 is a well-characterized tumor suppressor in some human cancers, but is frequently overexpressed without mutation in other cancers, including breast cancer. Here we demonstrate that BRG1 upregulates *de novo* lipogenesis and that this is crucial for cancer cell proliferation. Knockdown of BRG1 attenuates lipid synthesis by impairing the transcription of enzymes catalyzing fatty acid and lipid synthesis. Remarkably, exogenous addition of palmitate, the key intermediate in fatty acid synthesis, rescued the cancer cell proliferation defect caused by BRG1 knockdown. Our work suggests that targeting BRG1 to reduce lipid metabolism and, thereby, to reduce proliferation, has promise for epigenetic therapy in triple negative breast cancer.

## INTRODUCTION

Upregulation of lipogenic genes and overall lipogenesis are hallmarks of cancer [1]. Depending on the tumor type, tumor cells synthesize up to 95% of saturated and mono-unsaturated fatty acids (FA) *de novo* despite sufficient exogenous supply [2]. Lipogenic enzymes such as fatty acid synthase (FASN), acetyl-CoA carboxylase (ACC), and ATP citrate lyase (ACLY) that are involved in fatty acid biosynthesis and sterol regulatory element binding protein 1 (SREBP1), the master regulator of lipogenic gene expression, are overexpressed in a number of cancers including breast, prostate, ovarian, lung, and colon [3–6]. Several lines of evidence suggest that activation of the *de novo* fatty acid synthesis pathway is required for carcinogenesis [1, 7, 8]. For example, elevated levels of FASN, the major enzyme responsible for fatty acid biosynthesis, are correlated with poor prognosis in breast cancer patients [1, 7]. Increases in both FASN expression and activity are observed early in oncogenesis and correlate with cancer progression, with FASN-overexpressing tumors exhibiting more aggressive phenotypes [1]. Chemical or RNAi-mediated inhibition of key enzymes involved in fatty acid synthesis, including FASN, ACC and ACLY, reduces cell proliferation, induces apoptosis of cancer cells and retards the growth of human tumors in mouse xenograft models [1, 9–13].

Whereas various tumor types display increased endogenous fatty acid biosynthesis irrespective of extracellular lipid availability, most normal cells, even those with comparatively high proliferation rates, preferentially use dietary/exogenous lipids for synthesis of new structural lipids [1, 12]. We sought to investigate how lipogenic pathways are re-wired in cancer. Mammalian SWI/SNF complexes are evolutionarily conserved, multisubunit enzymes that mobilize nucleosomes and remodel chromatin using the energy of ATP hydrolysis [14–16]. These enzymes are important in DNA replication and repair, cell growth control, maintenance of pluripotency, and promotion of cell lineage differentiation. Increasing evidence supports an important role for human SWI/SNF enzyme subunits in cancer development [17, 18]. Meta-analyses of cancer genome-sequencing data estimates that nearly 20% of human cancers harbor mutations in one or more SWI/SNF genes [17–20]. We and others reported that knockdown of BRG1 reduces cell proliferation in both breast epithelial and cancer cells *in vitro* [21–23] and attenuates tumor growth in a xenograft model [21, 22]. However, the underlying mechanisms remained unknown. Here we report that BRG1 directly regulates triple negative breast cancer cell proliferation via regulation of lipogenic pathways. Knockdown of BRG1 decreased *de novo* lipid synthesis in breast cancer cells, but not in breast epithelial cells, with concomitant reduction in cell proliferation. BRG1 knockdown significantly reduced lipogenic gene expression. Chromatin immunoprecipitation analysis revealed that BRG1 was bound to sequences at lipogenic genes. Re-introducing BRG1 largely restored FASN and ACC expression, *de novo* lipid synthesis and cell proliferation. Supplementing the cell media with exogenous palmitate completely restored cell proliferation in BRG1 knockdown cells, thereby demonstrating a causal link between lipid synthesis and cancer cell proliferation and identifying a novel mechanism by which lipogenic signaling is crucial for cancer cell growth.

## RESULTS

### Reduction of BRG1 in cancer cells attenuated *de novo* lipid synthesis

**Figure 1:**
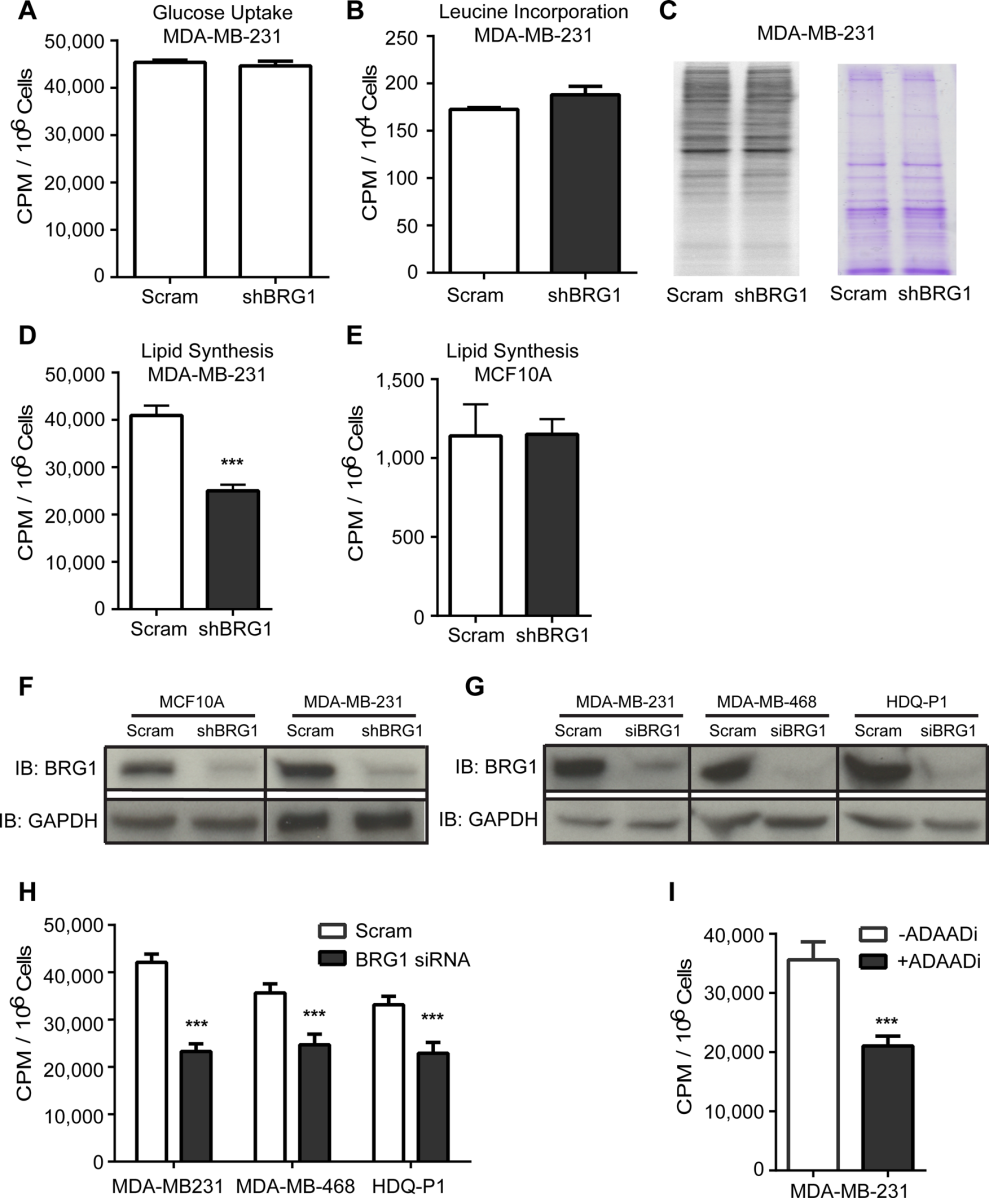
BRG1 knockdown reduced *de novo* lipid synthesis in triple negative breast cancer cells but not in MCF-10A mammary epithelial cells (**A**) MDA-MB-231 cells expressing either a scrambled sequence shRNA or an shRNA targeting BRG1 were incubated with ^14^C-glucose and incorporation of radioactivity into cells was measured to determine glucose uptake. There was no significant change. (**B**) BRG1 knockdown cells also had no change in the rate of protein synthesis compared to control cells as measured by ^3^H-leucine incorporation. (**C**) Phosphorimage of the blot of ^35^S-labeled protein extracted from control and BRG1 knockdown cells (left) and Coomassie brilliant blue staining of the blot (right). (**D**) BRG1 knockdown cells had a decrease in *de novo* lipid synthesis as measured by ^14^C-acetate incorporation in total extracted lipids. (**E**) MCF-10A cells expressing a scrambled sequence shRNA or shRNA targeting BRG1 were incubated with ^14^C-acetate and incorporation of radioactivity into extracted total lipids was measured. BRG1 knockdown did not cause a significant decrease in lipid synthesis. (**F**) Western blot analysis verified the shRNA-mediated knockdown of BRG1 in MDA-MB-231 and MCF-10A cells. (**G**) Western blot analysis verified the siRNA-mediated knockdown of BRG1 in MDA-MB-468 and HDQ-P1 cells. (**H**) Three triple negative breast cancer cell lines were treated with scrambled sequence siRNA or a cocktail of siRNAs targeting BRG1 and ^14^C-acetate incorporation into extracted total lipids was measured. (**I**) A small molecule inhibitor (ADAADi) of BRG1 inhibited *de novo* lipid synthesis in MDA-MB-231 cells. Each data point represents the mean of 3 independent experiments performed in triplicate. Error bars are standard deviations. ***P < 0.001.

One of the most conserved features of all cancers is the reprogramming of cellular metabolism in favor of biosynthetic processes that support high proliferation rates and survival in the tumor microenvironment [24]. To support unlimited growth, cancer cells exhibit higher rates of glucose metabolism, protein synthesis and *de novo* lipid synthesis [25, 26]. We surveyed these pathways by metabolic labeling in MDA-MB-231 triple negative breast cancer cells in the presence of a scrambled sequence shRNA or shRNA targeting BRG1 [21, 22, 27]. Glucose uptake and protein synthesis were not affected in MDA-MB-231 BRG1 knockdown cells (Figure 1A–1C). Interestingly, *de novo* lipid synthesis was reduced by 40% in the MDA-MB-231 BRG1 knockdown cells (Figure 1D) but not in MCF-10A breast epithelial cells expressing the same shRNA against BRG1 (Figure 1E). Western blot analysis confirmed the knockdown of BRG1 in both cell lines (Figure 1F). This observation was reproduced in other triple negative breast cancer lines (MDA-MB-468 and HDQ-P1) that were treated with a previously validated pool of siRNAs targeting BRG1 [22, 27] (Figure 1G–1H). ADAADi (Active DNA-dependent ATPase A Domain inhibitor), a minor product generated by the bacterial APH (3′)-III enzyme that encodes for aminoglycoside resistance, inhibits the ATPase activity of the SWI2/SNF2 family of ATPases [28, 29] and increases the chemosensitivity of triple negative breast cancer cells to clinically relevant therapeutic drugs [30]. Pharmacological inhibition of the BRG1 ATPase domain by ADAADi in MDA-MB-231cells also decreased *de novo* lipid synthesis (Figure 1I). Collectively, the data show a role for BRG1 in promoting *de novo* lipid synthesis in triple negative breast cancer cells.

### BRG1 upregulates lipogenic gene expression in triple negative breast cancer cells

**Figure 2:**
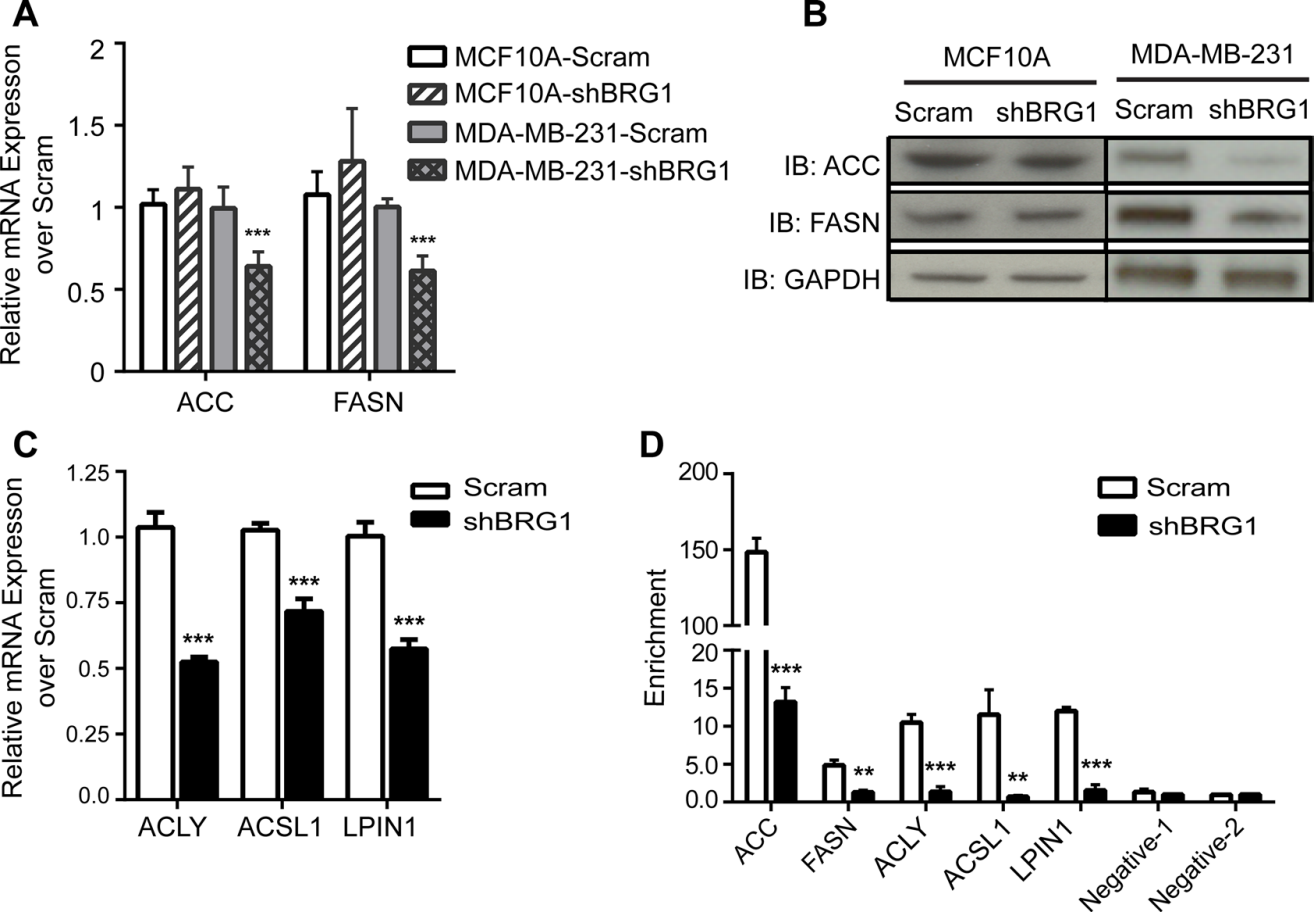
BRG1 was required for the expression of genes involved in fatty acid and lipid synthesis (**A**) mRNA levels of ACC and FASN in MDA-MB-231 and MCF-10A cells expressing either a scrambled sequence shRNA or an shRNA targeting BRG1 were measured by qPCR. (**B**) Western blot analysis measuring protein levels of ACC and FASN in MDA-MB-231 and MCF-10A cells expressing a scrambled sequence shRNA or shRNA targeting BRG1. (**C**) mRNA levels of ACLY, ACSL1 and LPIN1 in MDA-MB-231 cells expressing either a scrambled sequence shRNA or an shRNA targeting BRG1 were determined by qPCR. (**D**) ChIP experiments with MDA-MB-231 cells expressing either a scrambled sequence shRNA or an shRNA targeting BRG1 demonstrated that BRG1 binds to sequences upstream of each gene. Negative control sequences 1 and 2 are within the coding sequences of the ACC and FASN genes, respectively. Each dataset represents the mean of 3 independent experiments performed in triplicate. Error bars are standard deviations. **P < 0.01, ***P < 0.001.

The reduction in *de novo* lipid synthesis in BRG1 knockdown breast cancer cells was directly caused by reduction of lipogenic gene expression. *De novo* fatty-acid synthesis involves two key enzymes, ACC and FASN. ACC carboxylates acetyl-CoA to form malonyl-CoA, and the malonyl-CoA product is subsequently converted by FASN to palmitate, a precursor for longer-chain fatty acids [31]. As shown in Figure 2A, mRNA levels of ACC and FASN were down-regulated after BRG1 knockdown in breast cancer cells but not in non-tumorigenic MCF-10A mammary epithelial cells. The protein levels of these enzymes were also reduced by BRG1 knockdown in breast cancer cells, but not in non-tumorigenic MCF-10A mammary epithelial cells (Figure 2B).

The expression of other genes involved in the synthesis or metabolism of lipids was also regulated by BRG1 (Figure 2C). Elevated glucose catabolism produces an excess of the glycolytic end-product, pyruvate. Most excess pyruvate is converted to lactate, but some is converted to acetyl-CoA by ATP citrate lyase (ACLY) and can be used in *de novo* fatty-acid synthesis. Therefore, ACLY is the link between the metabolism of carbohydrates and the production of fatty acids and represents an important step in fatty acid biosynthesis [32]. Long-chain acyl CoA synthetase (ACSL) catalyzes the first step in fatty acid activation for intracellular metabolism by converting long-chain fatty acids into acyl-CoA thioesters [33, 34]. ACSL1 is the best-studied and the major ACSL isoform, is highly expressed in major energy-metabolizing tissues, and plays a key role in lipid biosynthesis and fatty acid degradation [35]. Lipin-1 (LPIN1) is a magnesium-dependent phosphatidic acid phosphohydrolase that catalyzes the penultimate step in triglyceride synthesis including the dephosphorylation of phosphatidic acid to yield diacylglycerol [36]. The expression of all these genes was decreased by BRG1 knockdown (Figure 2C).

We next showed that BRG1 directly bound to these genes having BRG1-dependent expression. We predicted BRG1 binding sites based on BRG1 ChIP-seq data from K562 and HeLa cells and global H3K27Ac and DNase I hypersensitivity analyses [37] and used these for PCR primer design within the region 1.5 Kb upstream of the TSS of the ACC, FASN, ACLY and ACSL1 genes (Supplementary Figure 1). There were no active transcriptional marks in sequences upstream of the LPIN1 TSS, however, there were multiple active marks in the first intronic region of this gene, allowing the design of PCR primers in this region (Supplementary Figure 1). Chromatin immunoprecipitation (ChIP) analysis detected BRG1 binding at these lipogenic genes in control cells that was markedly decreased in BRG1 knockdown cells (Figure 2D). Additional ChIP controls are shown in Supplementary Figure 2. These data suggest that BRG1 directly and transcriptionally regulates lipid biosynthesis and metabolism pathways in these breast cancer cells.

### Reduction in *de novo* lipid synthesis impaired cancer cell proliferation

**Figure 3:**
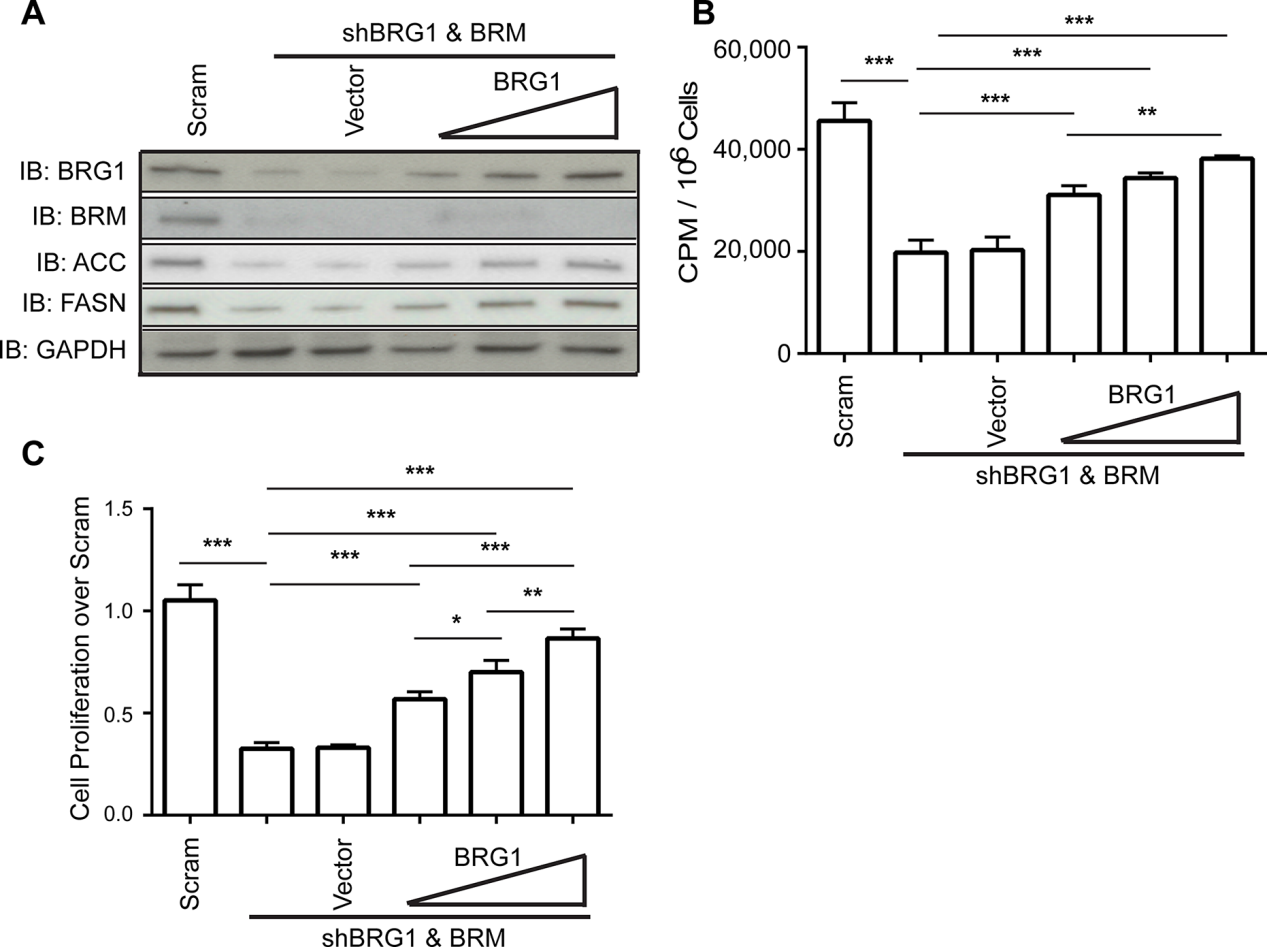
Restoration of BRG1 expression in cells depleted for BRG1 partially rescued the decrease in *de novo* lipid synthesis and cell proliferation (**A**) Western blot analysis showing that re-expression of BRG1 partially restored ACC and FASN protein levels. (**B**) Re-expression of BRG1 partially reversed the inhibition of *de novo* lipid synthesis caused by loss of BRG1. (**C**) Re-expression of BRG1 partially reversed the inhibition of cell growth caused by loss of BRG1. Each dataset represents the mean of 3 independent experiments performed in triplicate. Error bars are standard deviations. *P < 0.05, **P < 0.01, ***P < 0.001.

To further confirm that BRG1 is the direct cause for the reduction in lipid biosynthesis, a complementary experiment was performed to restore BRG1 expression in the knockdown cells. Re-expression or over-expression of wildtype or catalytically inactive BRG1 is negligible in some cell types if Brahma (BRM), the closely related ATPase that can act as the catalytic subunit in mammalian SWI/SNF complexes in a manner that is mutually exclusive with BRG1, is expressed [38–40]. In addition, we have previously shown that knockdown of either BRG1 or BRM in triple negative breast cancer cell lines results in increased expression of the remaining ATPase [22] and that both BRG1 and BRM contribute to triple negative cell proliferation [30]. Therefore we re-established BRG1 expression in MDA-MB-231 cells after expressing shRNA against both BRG1 and BRM [22] by transient transfection with plasmid vectors. Re-expression of BRG1 in this double knockdown background resulted in increasing levels of FASN and in a dose-dependent manner (Figure 3A), as well as a concomitant increase in *de novo* lipid synthesis (Figure 3B). As we previously reported [22], re-expression of BRG1 in double knockdown cells caused only a partial rescue in cell proliferation (Figure 3C). This is expected since we have shown that BRM also contributes to cell proliferation [30]. These data show that BRG1 regulates both *de novo* lipid synthesis and cell proliferation.

**Figure 4:**
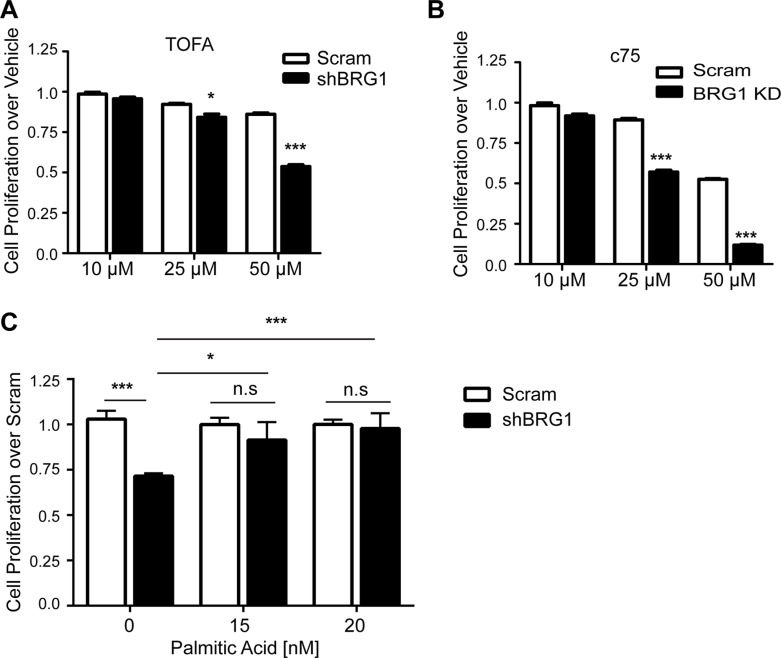
Fatty acid levels regulated breast cancer cell proliferation (**A**–**B**) BRG1 knockdown rendered cells more sensitive to growth inhibition by the ACC inhibitor TOFA or the FASN inhibitor c75. (**C**) Addition of palmitic acid to the cell culture media completely reversed the growth inhibition caused by BRG1 knockdown. (**D**) Addition of palmitic acid did not affect FASN or ACC expression in control or in BRG1 knockdown cells. Each dataset represents the mean of 3 independent experiments performed in triplicate. Error bars are standard deviations. *P < 0.05, ***P < 0.001, n.s. not significant.

We reasoned that, as an upstream regulator of key lipogenic enzymes, depletion of BRG1 should also increase the sensitivity of cells to fatty acid synthesis inhibitors. 5-tetradecyloxy-2-furoic acid (TOFA) is an inhibitor of ACC [41]. When TOFA was added to cells, the decrease in viable cell number was larger after BRG1 knockdown than in control cells (Figure 4A). The FASN inhibitor c75 [42] decreased cell viability in control cells, underscoring the need for *de novo* lipid biogenesis in highly proliferative tumor cells. As shown in Figure 4B, c75 potency was significantly enhanced in BRG1 knockdown cells compared to control cells. ACC and FASN have been reported to be essential to cancer cell survival, and knocking down either ACC or FASN dramatically decreases cancer cell proliferation [9, 43, 44]. After reducing BRG1 levels, breast cancer cells showed increased sensitivity to both inhibitors.

Since the end product of FASN and common intermediate in all *de novo* fatty acid synthesis is palmitate, we asked if the decrease in palmitate production was responsible for the impaired cell proliferation in cancer cells with BRG1 knockdown [22]. Following BRG1 knockdown, the cell culture medium was replaced with medium containing increasing doses of nonfat BSA-conjugated palmitic acid, and cell proliferation was measured. As shown in Figure 4C, addition of exogenous palmitic acid in the culture medium completely rescued the cell proliferation defect in BRG1 knockdown cells but had no effect on control cells. This result shows that BRG1-dependent contributions to *de novo* lipid synthesis in turn regulate the rate of the breast cancer cell proliferation.

Addition of palmitate did not affect ACC or FASN expression in either control of BRG1 knockdown cells (Figure 4D). Thus BRG1 function is upstream of ACC and FASN expression, which is upstream of palmitate production and *de novo* lipid synthesis, which are upstream of cell proliferation. The data do not exclude possible feedback signals whereby the altered proliferation rate also affects *de novo* lipid synthesis. Regardless, the results demonstrate that decreased cell proliferation in BRG1 knockdown cells can be attributed to the decrease in expression of the key metabolic enzymes for fatty acid synthesis.

## DISCUSSION

One of the hallmarks of cancer is elevated *de novo* fatty acid synthesis [45]. Clinical and basic scientific investigation has shown that human cancers synthesize fatty acids via the *de novo* fatty acid synthesis pathway even when exogenous fatty acids are abundant, seemingly independent of the regulatory signals that control fatty-acid synthesis in normal cells. The role of FASN in normal human biology includes energy storage from excess carbohydrates to fat in liver and adipose tissue and specialized functions that facilitate lactation in the breast and reproduction in endometrium and decidua [46–48]. FASN expression during these processes is strictly regulated by nutrition and hormonal levels [49]. In contrast, FASN is highly expressed in many cancers and precancerous lesions. In this context, the expression of FASN is independent of nutrition, and in many cases, it appears independent of hormonal regulation [45]. Despite its promise as a target for anti-cancer therapeutics [1, 12, 50, 51], the mechanism by which FASN is dysregulated in cancer is unknown. Similarly, two enzymes catalyzing rate limiting steps upstream of FASN in *de novo* fatty acid synthesis, ACC and ACLY, are often dysregulated in cancer and have been proposed as breast cancer therapeutic targets [52–54].

BRG1 is a known epigenetic regulator of chromatin structure and gene expression that may also play an architectural role in gene organization [55]. We show here that loss of BRG1 attenuated FASN, ACC, and ACLY expression and impaired *de novo* lipid synthesis in breast cancer cells with a concomitant decrease in cell proliferation. Importantly, this phenomenon was only seen in cancer cells and not in non-tumorigenic mammary epithelial cells. Re-introducing BRG1 to BRG1- and BRM-depleted cancer cells increased FASN and ACC expression, increased *de novo* lipid synthesis, and partially restored cell proliferation. When the culture medium was supplemented with palmitic acid, the end product of FASN and the key intermediate in the synthesis of longer chain and desaturated fatty acids, cell proliferation in BRG1 knockdown cells was completely rescued. The complete rescue in cells where only BRG1 was knocked down and BRM was present suggests that BRG1 is the SWI/SNF ATPase predominantly responsible for regulation of these metabolic pathways. Thus our results provide the first evidence of a direct relationship between BRG1, lipogenic enzyme transcriptional control, *de novo* fatty acid synthesis, and cell proliferation.

We and others have demonstrated that BRG1 levels are elevated in primary breast cancer [22, 23]. Our results here show that the elevated levels of lipid synthesis found in breast cancer are dependent on BRG1 acting to upregulate the transcription of FASN, ACC, ACLY and other genes involved in fatty acid synthesis. BRG1 is therefore an epigenetic link between breast cancer cell proliferation and fatty acid synthesis, but it is likely to be only the first factor identified in a novel regulatory circuit up-regulating lipogenic enzyme expression in cancer with downstream consequences on cell proliferation. Further investigation of BRG1 and the BRG1-interacting factors of this circuit will elucidate mechanisms by which cancer cells rewire signaling pathways controlling *de novo* lipid biosynthesis.

### Strategically targeting BRG1 for cancer therapy

BRG1 function in cancer appears to be context dependent. It is mutated in lung and other cancers [56, 57], and cancers featuring loss of the SNF5/INI1 subunit may require BRG1 [18], thereby suggesting the potential of targeting BRG1 to treat such tumors [58, 59]. In addition, BRG1 is upregulated with little evidence of mutation in primary breast and prostate tumors, in melanoma and neuroblastoma, and in pancreatic, gastric, and colorectal carcinomas [22, 23, 60–67]. Therefore, developing small molecule inhibitors that interfere with BRG1 function or that fine-tune the expression of BRG1 back to physiological levels might provide therapeutic benefits. Indeed, we have recently demonstrated that knockdown of BRG1 or use of a BRG1 inhibitor sensitizes triple negative breast cancer cells to commonly used chemotherapeutic agents, perhaps via modulation of ABC transporter expression [30].

The data presented here show that BRG1 is specifically required for fatty acid and lipid synthesis in triple negative breast cancer cells but not in mammary epithelial cells. This specificity increases the promise for BRG1- based therapies in triple negative breast cancer compared to treatment approaches that non-specifically target fatty acid and lipid synthesis. We conclude that targeting BRG1 and its cooperating factors are a promising and novel strategy for attacking both cancer cell proliferation and cancer metabolism.

## MATERIALS AND METHODS

### Cell culture

MCF-10A cells from the Karmanos Cancer Institute (Detroit, MI) were maintained in monolayer as described [68]. MDA-MB-231 cells were obtained from T. Guise [69]. MCF-10A cells inducibly expressing shRNA targeting BRG1 or a control shRNA were previously described [21]. MDA-MB-231 cells inducibly expressing shRNA targeting BRG1, BRG1 + BRM, or a control shRNA were previously described [22]. MDA-MB-468 cells were obtained from Professor Hong Zhang (UMass Medical School). HDQ-P1 cells were purchased from the Leibniz Institute DSMZ-German Collection of Microorganisms and Cell Culture (Braunschweig, Germany). MDA-MB-231, MDA-MB-468 and HDQ-P1 cells were maintained in DMEM containing 10% FBS and Penicillin/Streptomycin. Doxycycline-inducible knockdown was performed as described [21, 22, 27] using 500 ng/ml doxycycline for MCF-10A cells and 100 ng/ml doxycycline for MDA-MB-231 cells. siRNA-mediated knockdown of BRG1 was performed using the validated pool of siRNAs and methods described previously [22, 27].

The identities of the breast cancer cell lines were authenticated by Short Tandem Repeat profiling at the Genetic Resources Core Facility, Johns Hopkins School of Medicine, Institute of Genetic Medicine. MCF-10A cells were similarly authenticated at the University of Vermont Cancer Center.

### Reagents

Doxycycline, palmitic acid, TOFA, c75, MTT (3-(4, 5-dimethylthiazolyl-2)-2,5-diphenyltetrazolium bromide), and anti-GAPDH antibody were purchased from Sigma (Sigma-Aldrich, St. Louis, MO). FASN (C20G5) and acetyl-CoA carboxylase (C83B10) antibodies were purchased from Cell Signaling (Cell Signaling Technology, Inc., Danvers, MA, USA). Anti-rabbit and anti-mouse IgG, and HRP-linked antibodies were from GE (GE Healthcare Life Science, Pittsburgh, PA, USA). BRG1 antiserum [70] was used in western blot and ChIP experiments. The BRM antibody (ab15597) used in western blots was purchased from Abcam (Cambridge, MA, USA). D-^14^C-Glucose, ^3^H-Leucine, ^35^S-Methionine and [2–^14^C] acetic acid were purchased from PerkinElmer (PerkinElmer Life Sciences, Waltham, MA, USA). ADAADi was prepared as described [28].

### Proliferation assays

Five thousand cells were seeded in each well of 96-well plates, and treated as indicated in each figure legend the following day. MTT solution at a final concentration of 5 ug/mL was added to each well and samples were incubated for 4 hours. The media was subsequently removed and the plates were air-dried. One hundred microliters of DMSO were added to each well and incubated for 30 minutes at room temperature with gentle shaking. Absorbance was measured at OD540 in a Synergy H4 Hybrid microplate reader (Bio Tek, Winooski, VT).

### Glucose uptake assay

Cells were pulse labeled with 1 μCi ^14^C-glucose for 5 minutes, and then washed 3 times with PBS. Cells were trypsinized and counted. One million cells were lysed by adding 200μL 0.2N NaOH for 15 min. The cell lysate were transferred to a scintillation vial, and radioactive signal was measured in a scintillation counter (Beckman Coulter LS6500).

### Protein synthesis assays

Protein synthesis rate was determined by labeling cells with 1 μCi ^3^H-Leucine for 4 h. After three washes in ice cold PBS, cells were lysed with PBS containing 2% SDS before scintillation counting. All readings were normalized by cell number determined from parallel plates. For other protein synthesis assays, cells were labeled with 10 μCi^35^S-Methionine overnight, washed 3 times with ice cold PBS, and then lysed with PBS containing 1% NP-40. Radiolabeled proteins (2 μg) were run on SDS-PAGE 4–20% gradient gels before transfer to a PVDF membrane. Radioactivity was measured by Phosphoimager and checked for equal protein loading by staining the membrane with Coomassie Brilliant Blue.

### *De novo* lipid synthesis assay

In experiments with ADAADi, cells were pre-treated with 2 μM ADAADi for 48 h prior to addition of radiolabeled acetate. Breast cancer cells were labeled with 1 μCi /mL ^14^C-acetate for 1 h. MCF-10A cells were labeled with 5 μCi /mL ^14^C acetate for 1 h. After removal of the labeling medium, cells were washed 3 times with PBS and then cultured in fresh medium for 24 h. At the end of the incubation, cells were trypsinized and counted. One million cells were used for lipid extraction. Total lipids were extracted as described [71], transferred to a scintillation vial and counted in a scintillation counter (Beckman Coulter LS6500).

### c75, TOFA and palmitic acid treatment

MDA-MB-231 SCRAM and shBRG1 cells were plated in 96-well plates at a density of 3,000 cells/well and treated with 0.1 μg/ml doxycycline or with vehicle for 48 h. Reagents were added to the cell culture medium at 0, 15, or 20 nM for 24 h (palmitic acid) or at 10, 25, or 50 μM for 72 h (c75, TOFA). To assess cell proliferation, 10 μL of MTT reagent was added to each well and incubated for 4 h prior to an MTT assay.

### Western blotting

Cells were washed, trypsinized, and counted and whole cell lysates from one million cultured cells were prepared by lysis in 200 μL of 1× Laemmli sample buffer and boiled for 5 min. 10 μL of lysate was separated on SDS-polyacrylamide gels (4–20% gradient) and transferred to PVDF membrane. Membranes were blocked in 5% non-fat dry milk in PBS, incubated with primary antibodies overnight at 4°C. Following repeated washing in 5% milk/PBS, membranes were incubated with secondary antibody conjugated to HRP for 1 hour at room temperature, washed repeatedly with 5%milk/PBS, and developed using Amersham ECL Western Blotting Detection Reagents and Amersham Hyperfilm ECL (GE LifeScience).

### RT-qPCR

One million cells were used for extraction of total RNA using RNeasy Plus following manufacturer's instructions (Qiagen Inc., Valencia, CA, USA). cDNA synthesis was accomplished using a SuperScript III kit (Invitrogen, San Diego, CA, USA). Gene expression was measured by real time qPCR using following primers. ACC alpha (ACACA) forward 5′-ATGCG GTCTATCCGTAGG-3′, reverse 5′-GGTGTGACCATGA CAAC-3′; FASN, forward 5′-GTTTGATGCCTCCTTCTTC-3′, reverse 5′-CGGAG TG AATCTGGGTTG-3′; ACLY forward 5′- CCCAAGTCCAAGATCCCTGC-3′, reverse 5′- TCGTCTCGG GAGCAGACATA-3′; ACSL1 forward 5′- GGAAGAGCCAACAGA CGGAA-3′, reverse 5′- CATTGCTCCTTTGGGGTTGC-3′; LPIN1 forward 5′- GAGGCAGAC AGCACCATACA-3′, reverse 5′- GGCTAACTGCCCCACGTAAT-3′.

### Chromatin immunoprecipitation (ChIP)

ChIP was performed as described previously [38] with modifications. Cells were cooled to room temperature before crosslinking which was done by replacing the media with ice-cold growth medium containing 3.7% formaldehyde for 40 min at 4°C. Each ChIP reaction used 5 μg of BRG1 antiserum [70] or control IgG (Millipore, Billerica, MA) and 50 μg of chromatin extract. Primers used for measuring BRG1 binding at those promoters measured by qPCR are listed below: ACC alpha (ACACA), forward 5′- GTCCCACCCCGTAAGGATTT -3′ (−537 ~ −556 bp to TSS), reverse 5′- GGCGCTAGCTCCAAACTAAC -3′ (−708~−727 bp to TSS);

FASN, forward 5′- CTCCCGAGTGATTCCTCGAA -3′(−1086 ~ −1105 bp to TSS), reverse 5′- CTCAAAGT AGGACACGCAGC -3′ (−1233 ~ −1252 bp to TSS);

ACLY, forward 5′- GTAAGCAAGTGGGGCTAGG AG -3′ (−570 ~ −590 bp to TSS), reverse 5′CTTCGCT GGAATCTCGCATTG -3′ (−665~ −684 bp to TSS);

ACSL1, forward 5′- CCAGACTGCCTCGGA TTTCATA -3′ (−130 ~ −151 bp to TSS), reverse 5′- GGCGGTCCAATGTACCCTT

-3′ (−172 ~ −191 bp to TSS);

LIPIN1, forward 5′-TGCAGCCCATTTCCTGG ATT-3′ (+66,769 bp ~ +66,788 bp to TSS), reverse 5′-GAGGAAGGAGGGGCTGAGTA-3′ (+66,842 bp ~ (+66,861 bp to TSS).

Genomic region (chr17:35,716,490-35,716,996) near the ACACA promoter was used as a negative control for BRG1 binding, forward 5′-ATACATGC TGGATCCTGGCG-3′, reverse 5′-GGACGGGAAGCA TTCTCCAA-3′. Genomic region (chr17:80,055,818-80,056,106) near the FASN promoter served as an additional negative control for BRG1 binding, forward 5′-CTCCGAAGGGGCACGAAC-3′, reverse 5′-TCCTC ATCCTCCGCTCTCG-3′. Meanwhile, ABCC3 gene expression was not affected by depletion of BRG1 in our previous work [30]. Therefore BRG1 binding at this locus was used to serve additional negative control, with primer sequences as following: forward 5′- ATTCAGGAGGGAGCTTTGCC-3′ (+4,555 bp ~ +4,754 bp to TSS), reverse 5′- CCATTTCCCTGTCTGGGGAC-3′(+4,589 bp ~ +4,608 bp to TSS). RNase polymerase II binding at this locus was used as positive control in the ChIP experiment.

### Statistical analyses

Quantified data represent the mean of three independent experiments performed in triplicate with standard deviation (S.D). Statistical relevance was evaluated using GraphPad Instat two-tail *P* value student test (Graphpad Software, Inc., La Jolla, CA).

## SUPPLEMENTARY MATERIALS FIGURES


