# Supplementary material for: The BRG1 chromatin remodeling enzyme links cancer cell metabolism and proliferation
Source: Oncotarget. 2016 May 20;7(25):38270–81. doi: 10.18632/oncotarget.9505 (PMC5122388; doi:10.18632/oncotarget.9505)
Supplement: Supplementary file 1 [file oncotarget-07-38270-s001.pdf]

The BRG1 chromatin remodeling enzyme links cancer cell metabolism and proliferation

Supplementary Materials

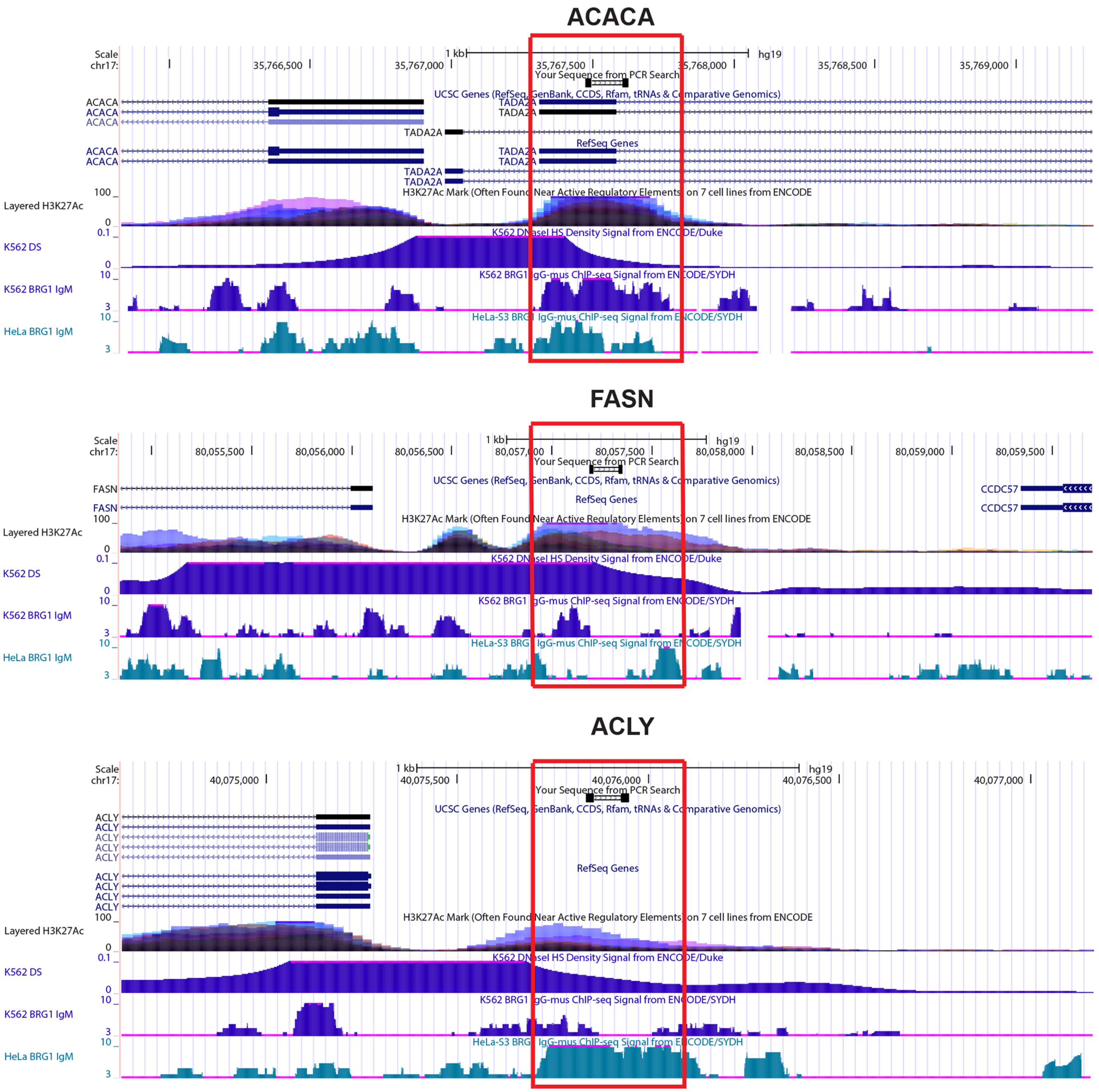

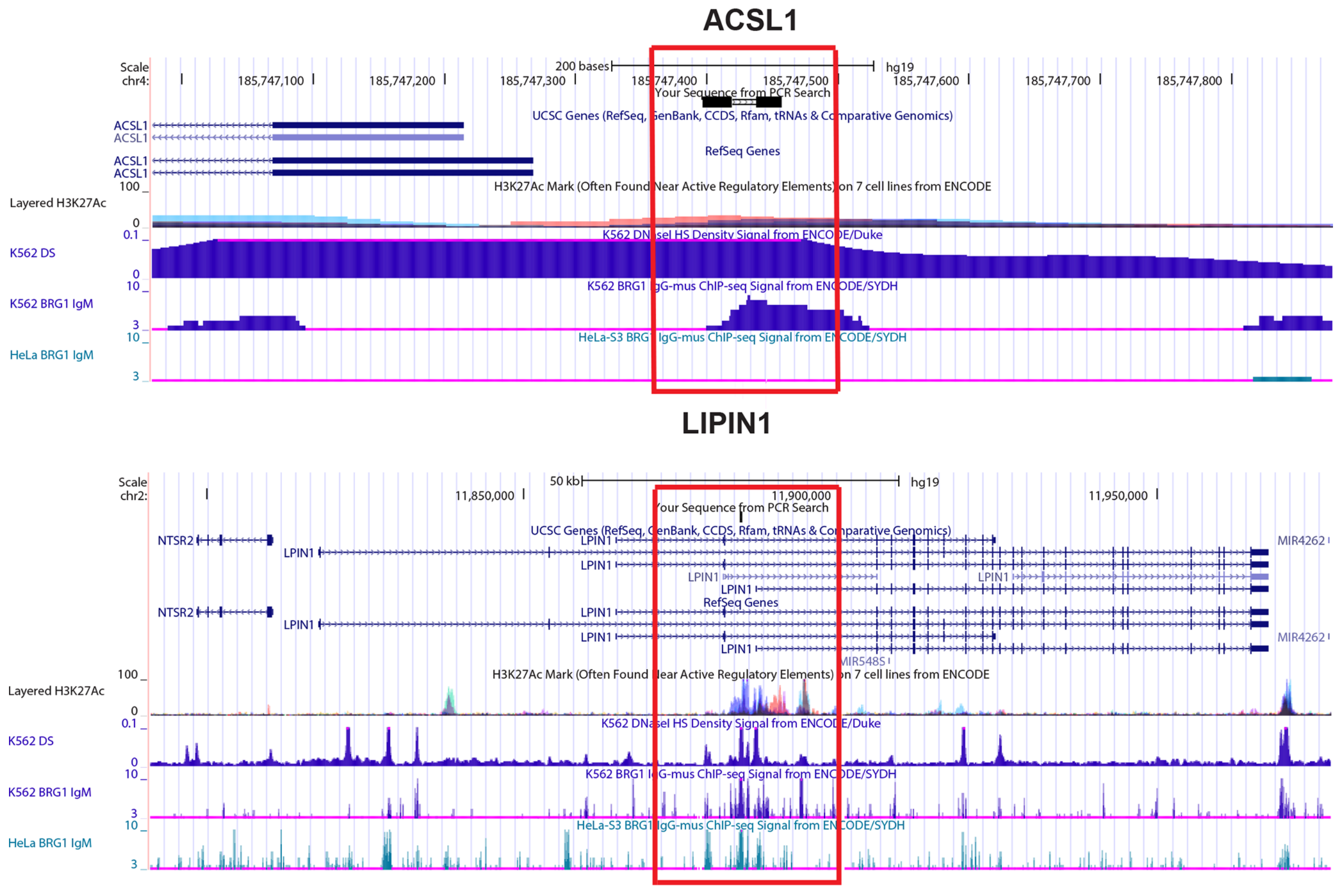

Supplementary Figure S1: Genomic Data for Chromatin Immunoprecipitation (ChIP) Primer Design. For each target gene we used ENCODE data to predict RNA transcripts (top rows), H3K27Ac sites (second rows), DNase I hypersensitivity sites (third rows), BRG1 ChIP-seq data from K562 cells (fourth rows), and BRG1 ChIP-seq data from HeLa cells (bottom rows). These data were used for PCR primer design; the red box in each panel contains the amplified sequence. For the ACC, FASN, ACLY and ACSL1 genes primers were chosen in a region 1.5 kb upstream of the TSS. There were no active transcriptional marks in sequences upstream of the LPIN1 TSS but there were multiple active marks in the first intronic region of this gene, allowing the design of PCR primers in this region.

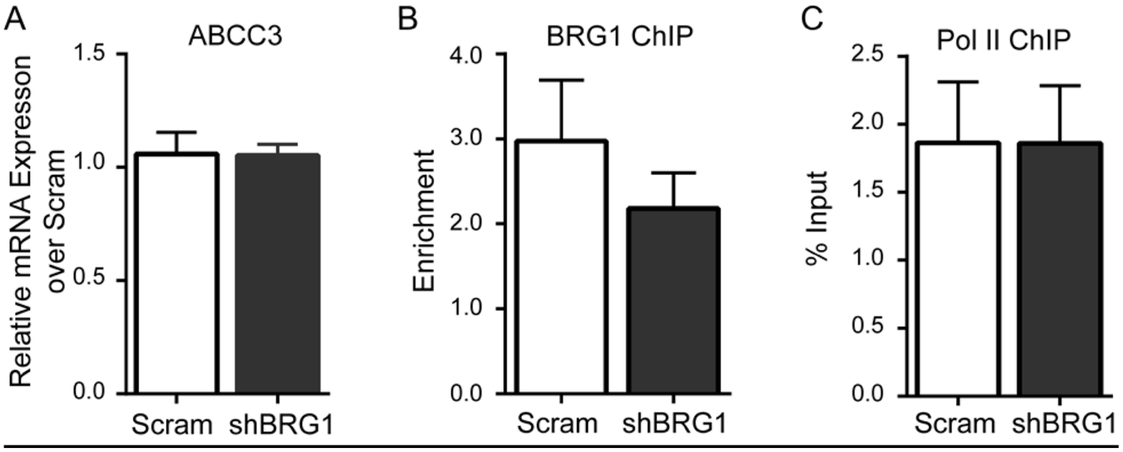

Supplementary Figure S2: Additional ChIP controls. ABCC3 is a member of the ABC transporter family that encodes energy-dependent transporters linked to efflux of chemotherapeutic drugs from cells (Gottesman and Ling. FEBS Lett. 2006, 580:998–1009; Holohan et al. Nat Rev Cancer. 2013, 13:714–726.). In a previous report, we determined that BRG1 binds to the ABCC3 locus but that basal levels of ABCC3 gene expression are not altered by BRG1 knockdown (Wu et al. Oncotarget. 2016, doi: 10.18632/oncotarget.8384). (**A**) Independent verification that BRG1 knockdown did not affect ABCC3 gene expression. Scram; cells expressing control shRNA. (**B**) ChIP measuring BRG1 binding to the ABCC3 locus in cells expressing control shRNA or shRNA targeting BRG1. The difference between the values was not statistically significant as measured by a two-tailed Students *t* test. (**C**) ChIP measuring RNA Pol II binding to the ABCC3 locus in both cell types. Each experiment shows the mean +/− standard deviation from three separate experiments each measured in triplicate.
